# Fisetin alleviates lipopolysaccharide-induced mastitis by inhibiting ferroptosis and modulating the gut microbiota

**DOI:** 10.1080/01652176.2026.2642789

**Published:** 2026-03-17

**Authors:** Chao Tong, Li Wang, Haojie Wen, Xinhui Yao, Jiang Tong, Ruya Zhang, Xiao Li, Yuqiang Xiang, Xuebing Wang

**Affiliations:** a College of Veterinary Medicine, Henan Agricultural University, Zhengzhou, PR China; b Ministry of Education Key Laboratory for Animal Pathogens and Biosafety, Henan Agricultural University, Zhengzhou, PR China; c Zhengzhou Key Laboratory of Research and Evaluation of Traditional Chinese Veterinary Medicine, Zhengzhou, PR China; d Henan Province Key Laboratory of Animal Food Pathogens Surveillance, Zhengzhou, PR China; e Henan Academy of Sciences, Zhengzhou, PR China; f Henan High Tech Industry Co., Ltd, PR China

**Keywords:** Ferroptosis, Oxidative stress, Bovine mastitis, Fisetin, Gut microbiota, Gut metabolites

## Abstract

Bovine mastitis poses an enormous challenge to the dairy industry. At present, dependence on antibiotic therapy has led to problems such as antibiotic-resistant bacteria and drug residues. Therefore, it is very important to seek alternative therapies for the treatment of bovine mastitis. Fisetin (FIS) with antioxidant and anti-inflammatory properties is found in various fruits and vegetables, although its regulatory role in mastitis treatment remains unclear. In the present study, we employed lipopolysaccharide (LPS)-induced cellular and animal mastitis models to investigate the regulatory effects of FIS on oxidative stress and ferroptosis pathways in mastitis treatment, utilizing techniques such as western blot, quantitative real-time PCR (qRT-PCR), immunofluorescence, hematoxylin-eosin (HE) staining, ROS, MDA, SOD, T-AOC, tissue total iron content analysis, mouse gut microbiota sequencing, and untargeted metabolomics. The results demonstrated that FIS modulates oxidative stress and ferroptosis pathways, leading to a reduction in intracellular and mouse mammary tissue inflammatory cytokines level. Furthermore, FIS treatment altered the gut microbiota structure and metabolites in LPS-induced mice, increasing short-chain fatty acid (SCFA) levels, which contributed to the restoration of the blood-milk barrier and alleviation of mastitis. This study could lead to novel therapeutic strategies for bovine mastitis based on traditional Chinese medicine.

## Graphical Abstract

**Figure:**
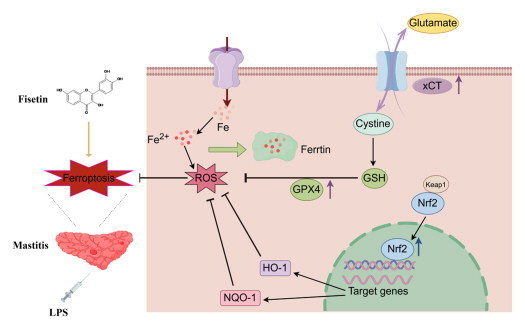

## 1. Introduction

Mastitis poses a significant challenge to the global dairy industry, as it can lead to heavy economic losses (Heikkilä et al. 2012). Mastitis also diminishes both the yield and quality of milk, potentially increasing the risks to human health (Paramasivam et al. 2023). Mastitis can be classified into four clinical forms, including peracute, acute, subacute, and chronic mastitis. Various environmental pathogens capable of inducing mammary gland damage have been identified, with *Escherichia coli* being a prominent causative agent and the lipopolysaccharide (LPS) it produces being a key virulence factor (Tong et al. 2025). Studies have revealed a substantial endotoxin reservoir predominantly comprising Gram-negative bacteria within the bovine gut (Li et al. 2021), especially the rumen. LPS is transported *via* the bloodstream to the mammary tissue where it activates the inflammatory response (Hu et al. 2022), which is characterized by pain and the upregulated expression of cytokines (Zaatout 2022), thereby representing an endogenous etiology.

Numerous studies have established correlations between mastitis and oxidative stress responses, and acute-phase proteins (APPs) have been identified as a potential biomarker for the detection of mastitis (Sadat et al. 2023). Oxidative stress is closely related to the generation and clearance of oxidation products; for instance, excessively high reactive oxygen species (ROS) levels can induce tissue and organ damage (Yang and Lian 2020). Adverse effects can occur if the accumulation of oxidation products surpasses a critical threshold; therefore, their timely elimination is crucial for maintaining redox homeostasis. Mechanistically, oxidation products activate nuclear factor erythroid 2-related factor 2 (Nrf2), which is commonly regarded as the primary regulator of redox homeostasis. Under normal physiological conditions, Nrf2 is ubiquitinated by the Kelch-like ECH-associated protein 1–RING-box protein 1–Cullin-3 ubiquitin ligase (Keap1–RBX1–CUL3) complex and subsequently degraded by the 26S proteasome. However, oxidation products promote the dissociation of Nrf2 from Keap1 and undergo translocation into the nucleus where it promotes the synthesis of antioxidants and regulates the transcription of downstream genes, such as nicotinamide adenine dinucleotide phosphate (NADPH) quinone oxidoreductase 1 (NQO-1) and heme oxygenase-1 (HO-1) (Tonelli et al. 2018; Romero-Durán et al. 2024). NQO-1 facilitates the excretion of quinones, thereby preventing their oxidation and the subsequent generation of ROS within an organism (Dinkova-Kostova and Talalay 2010), whereas HO-1, another critical antioxidant enzyme, degrades prooxidant heme groups (Drummond et al. 2019).

Emerging research has consistently demonstrated that the dynamic equilibrium in the initiation and progression of ferroptosis is related to the mechanisms of oxidative stress (Yang et al. 2022; Wang et al. 2023; Zhu et al. 2023). Glutathione (GSH), the most abundantly expressed endogenous antioxidant, interacts with glutathione peroxidase 4 (GPX4) to neutralize ROS (Harris and DeNicola 2020). GPX4 is a critical regulator of ferroptosis, and knockout of the GPX4 gene has been shown to induce ferroptosis in the most cells (Angeli et al. 2014). GSH synthesis is associated with solute carrier family 7 member 11 (SLC7A11) levels, and the SLC7A11 protein indirectly promotes GSH production. To maintain iron homeostasis, ferritin heavy chain (FTH) and ferritin light chain (FTL) function as iron storage proteins, promoting the oxidation of Fe^2+^ to Fe^3+^, thereby reducing intracellular concentrations of labile iron and maintaining cellular stability. Thus, targeting ferroptosis could represent a potential therapeutic approach for mastitis.

The administration of antibiotics remains the primary therapeutic avenue for mastitis; however, antibiotic resistance and the emergence of resistant bacterial strains (Algammal et al. 2020; Ma et al. 2021; Kløve et al. 2022), which has spurred the exploration of novel therapeutic agents and alternative treatment options (Ajose et al. 2022; Sindhu et al. 2024). The gut–mammary gland axis has emerged as a primary research focus, and the gut microbiota has been shown to significantly impact the pathogenesis of mastitis. In previous studies, microbial dysbiosis was shown to reduce the levels of short-chain fatty acids (SCFAs) in mice, and the subsequent administration of sodium propionate and sodium butyrate altered the blood–milk barrier and ameliorated *Staphylococcus aureus*-induced mastitis in mice (Hu et al. 2020). These findings suggest that increased levels of SCFAs and larger populations of beneficial bacteria or lower proportions of pro-inflammatory bacteria may alleviate mastitis either by strengthening the blood–milk barrier or by enhancing anti-inflammatory responses. Furthermore, an increasing number of studies have investigated the effects of low-cost and readily available traditional Chinese medicines in the pursuit of enhanced health outcomes (Mushtaq et al. 2018; Kovačević et al. 2022).

Fisetin (FIS), a flavonoid with diverse pharmacological effects, is a major component of the Chinese herb *Cotinus coggygria* and is present in various fruits, vegetables, and plants belonging to the Fabaceae and Anacardiaceae families (Matić et al. 2016). Stanislav Sukhikh et al. investigated the antibacterial and antioxidant enzyme activities of *Cotinus coggygria* extract. Their results showed that the extract had antibacterial activity against *E.coli*, *Staphylococcus aureus*, *Bacteroides vulgaris, Clostridium perfringens, and Clostridium enterotoxigenum*. The extract also exhibited antioxidant activity of 145.09 ± 7.25 mg AA/g (Sukhikh et al. 2021). Extensive studies have demonstrated that FIS exerts anti-inflammatory and antioxidant effects, while also inhibiting ferroptosis (Kashyap et al. 2019). FIS, a free radical scavenger, has been reported to inhibit tumor development in aflatoxin B1 (AFB1)-induced hepatocarcinoma in rats by modulating ROS levels to restore GSH and antioxidant enzyme concentrations (Maurya and Trigun 2016). FIS can also alleviate endometritis by inhibiting Toll-like receptor 4 (TLR4) and activating Nrf2/HO-1 (Jiang et al. 2021). Moreover, FIS has been shown to inhibit erastin- and glutamate-induced ferroptosis in *in vitro* models involving HT22 cells (an immortalized mouse hippocampal cell line) by reducing lipid peroxidation products (Yang et al. 2024). However, it remains unknown whether FIS can alleviate bovine mastitis. Therefore, this study aims to investigate the therapeutic efficacy of FIS against LPS-induced mastitis, as well as its effects on the gut microbiota and metabolites of mastitis-affected mice.

## 2. Materials and methods

### 2.1. Materials and reagents

Fisetin (WKQ-0000418, HPLC ≥98%) was purchased from Weikeqi (Sichuan, China), and LPS (L2880) was purchased from Sigma-Aldrich (St. Louis, MO). Antibodies against the following proteins were utilized in this study: GAPDH (Proteintech, 60004-1-Ig), Nrf2 (Proteintech, 16396-1-AP), IL-1β (Proteintech, 16806-1-AP), Keap1 Polyclonal antibody (Proteintech, 10503-2-AP), HO-1 (Proteintech, 10701-1-AP), xCT (Abways, CY7046), GPX4 (Abways, CY6959), FTH (Abclonal, A19544), FTL (Abclonal, A11241), NQO-1 (Abmart, T56710), TNF-α (Wanleibio, WL01581), and IL-6 (Wanleibio, WL02841). Malondialdehyde (MDA) assay kit (A003-1-1), Total Superoxide Dismutase (T-SOD) assay kit (A001-1-1), and Total antioxidant capacity assay kit (A015-2-1) were obtained from Nanjing Jiancheng Bioengineering Institute. The Tissue Iron Content Assay Kit (BC4355) and Reactive Oxygen Species Assay Kit (CA1410) were purchased from Solaibro. The Cell Counting Kit-8 (CCK-8) was purchased from TargetMol Chemicals Inc. (C0005), while Trizol (R401-01), HiScript II Q RT SuperMix for qPCR (+gDNA wiper) (R223-01), and ChamQ Universal SYBR qPCR Master Mix (Q711) were obtained from Vazyme.

### 2.2. Cell culture and grouping

MAC-T cells were a generous gift from Professor Wang Yueying of Henan Agricultural University, and were cultured in a 37 °C cell culture incubator with 5% CO_2_. The cell culture medium consisted of 90% DMEM, 10% serum, and 1% penicillin, streptomycin, and gentamicin. The grouping is as follows: Control, LPS, LPS+FIS, and FIS. Treatments commenced 12 h after cell passage. The LPS+FIS and FIS groups were treated with 4 μg/mL of FIS for 12 h, followed by a medium change to standard culture medium. Subsequently, the LPS and LPS+FIS groups were treated with 100 μg/mL of LPS for 12 h before proceeding to subsequent assays.The purpose of setting up the FIS group is to indicate its safety for MAC-T cells.

### 2.3. Establishment of animal models

Specific pathogen-free (SPF) KM mice, 8 weeks of age and weighing over 30 g, were obtained from Liaoning Changsheng biotechnology co., Ltd. 30 female mice and 15 male mice were followed a one-week acclimation period, the mice were co-housed and allowed to breed for approximately 21 days. Lactating females (5–7 days postpartum) were randomly assigned to groups: Control, LPS, LPS+FIS (50 mg/kg), LPS+FIS (100 mg/kg), and DEX, *n* = 6. The Control, LPS, and DEX groups were administered physiological saline *via* gavage. The FIS treatment groups received the corresponding concentrations of FIS *via* gavage. After 7 consecutive days of gavage, the DEX group received an intraperitoneal injection of 5 mg/mL DEX. Except for the Control group, other groups received LPS (0.2 mg/mL, 50 μL) along the mammary ducts of the fourth pair of mammary glands; the Control group received physiological saline as a control. After 24 h, the mice were euthanized, and blood was collected *via* the orbital sinus to obtain serum. Mammary gland tissue and fecal samples were simultaneously collected, part of the samples were fixed and stored at room temperature, while the other part was immediately frozen in liquid nitrogen and stored at −80 °C.

### 2.4. HE staining

Following the excision of murine tissue, it was fixed in 4% paraformaldehyde for a minimum of 24 h. The tissue underwent paraffin embedding, sectioning, dewaxing, staining, dehydration, permeabilization, and coverslipping. Subsequently, the samples were captured images using an optical microscope.

### 2.5. Cellular ROS detection

MAC-T cells were passaged into a 6-well plate, and treated with the experimental compounds. DCFH-DA was diluted to 10 μM in serum-free culture medium, then add 1 mL to each well. The plates were incubated at 37 °C for 30 min. The cells were then washed thoroughly by using serum-free medium to remove unbound probe. Then the images were captured using an inverted fluorescence microscope.

### 2.6. Immunofluorescence

Cellular immunofluorescence: Following cell processing, cells were fixed with pre-chilled methanol at 4 °C for 15 min, washed with PBS, permeabilized at room temperature for 10 min, and blocked with BSA solution for 60 min. Subsequently, primary antibody incubation was performed overnight. After washed with PBS, the cells were incubated with the corresponding fluorescent secondary antibody in the dark for 1.5 h. Following a 5-minute wash in the dark, nuclear staining was conducted for 15 min. After three washes with PBS, images were captured and preserved using an inverted fluorescence microscope.

Tissue immunofluorescence: Remove the tissue fixed in 4% paraformaldehyde and rinse it under running water for 24 h, followed by dehydration, tissue block embedding, sectioning, and dewaxing. Subsequently, the dewaxed tissue sections underwent antigen retrieval and were washed with PBS. Followed by overnight incubation with the primary antibody at 4 °C. On the second day, the sections were washed with PBS and incubated with the corresponding secondary antibody for 50 min. Finally, after nuclear staining of the sections with DAB chromogen solution, they were dehydrated and mounted with neutral resin. Images were captured using a laser scanning confocal microscope (LSM800, Carl Zeiss AG, Oberkochen, Germany).

### 2.7. Immunohistochemistry

Embedded tissue paraffin blocks were sectioned, dewaxed, and subjected to antigen retrieval, 3% hydrogen peroxide treatment, serum blocking at room temperature, overnight primary antibody treatment at 4 °C, and secondary antibody application at room temperature, followed by chromogenic development and coverslipping. Finally, images were captured using a laser scanning confocal microscope.

### 2.8. Determination of MDA, SOD, T-AOC, and tissue iron content

Following the kit instructions precisely, lyse cells with extraction buffer and a cell disruptor after cell processing. For animal tissues, weigh mouse mammary tissue and place it in pre-chilled extraction buffer. Homogenize with sterile steel beads in a homogenizer. Centrifuge at low temperature, and collect the supernatant for detection.

### 2.9. Western blot

Following cell processing, wash three times with PBS. Lyse thoroughly on ice for 30 min with the addition of pre-chilled RIPA buffer and a protease/phosphatase inhibitor cocktail. After sonication, centrifuge at low temperature and collect the supernatant. Determine protein concentration using a BCA assay kit. After mixing the supernatant with loading buffer, denature in a 100 °C water bath for 10 min. Cool to room temperature, load, and aliquot for storage at −20 °C. For animal tissues, weigh a section of mammary tissue, add RIPA buffer with protease and phosphatase inhibitors, homogenize, centrifuge, and collect the supernatant to determine the concentration before denaturation, aliquoting, and storage.

Perform polyacrylamide gel electrophoresis, loading 15–20 μg of protein per well. After electrophoresis, transfer the protein to PVDF membranes. Block with 5% non-fat milk at room temperature for 2 h. Incubate with the primary antibody overnight at 4 °C, followed by incubation with the secondary antibody at room temperature for two hours. Visualize protein bands using a chemiluminescence detection system (Tanon-5200, Shanghai Tianneng Life Sciences Co., Ltd.), and capture images for data analysis.

### 2.10. Quantitative real-time PCR (qRT-PCR)

After passaging and treating cells in 6-well plates, wash them with PBS buffer. Subsequently, add 1 mL of Trizol lysis buffer to each well to facilitate cell lysis. Extract RNA using the chloroform extraction method, dissolve the extract in sterile, nuclease-free water, and determine its concentration. Perform RNA reverse transcription strictly according to the Vazyme reverse transcription kit instructions. Subsequently, qRT-PCR was performed according to the Vazyme DNA Polymerase Mix protocol. Three biological replicates were established for each experimental group. Relative mRNA expression was calculated using the 2^(-ΔΔCT) method. Primer sequences are detailed in Table 1.

**Table 1.** Primer sequences of qRT-PCR.

Genes	Sequence (5′→ 3′)	Length(bp)	Serial number
*GAPDH*	F:GATGGTGAAGGTCGGAGTGAAC R:GTCATTGATGGCGACGATGT	100	NM_001034034.2
*Keap1*	F:AGTGGCGAATGATCACAGCA R:ATACAGTTGTGCAGGACGCA	70	NM_012289.4
*Nrf2*	F:AGAATAAAGTGGCTGCTC R:CTTCAAGATACAAGGTGCT	174	NM_001011678.2
*HO-1*	F:GGAGATCGAACGCAACAAG R:TCCTGGAGTCGCTGAACAT	171	NM_001014912.1
*NQO-1*	F:TTCAATCCCGTCATCTCC R:TTTGTTCGGCCACAATAT	130	NM_001034535.1
*GPX4*	F:TGTGGTTTACGGATCCTGGC R:CCCTTGGGCTGGACTTTCAT	182	NM_001346431.1
*SLC7A11*	F:GCCTTGTCCTACGCTGAACT R:GGCTGCAGGGCGTATAATGA	135	XM_024977578.2
*FTH*	F: TTGCCAAATACTTTCTTCAC R: TCCCAGTCATCACGGTCT	130	NM_174062.4
*FTL*	F:TATTTCGACCGCGACGATGT R:CACGTCCAGGAAGAGGGC	138	NM_174792.4

### 2.11. 16s rRNA gene sequencing analysis

The following groups were generated for subsequent intergroup comparisons: Control, LPS, and Fisetin. The Fisetin group is LPS+FIS (100 mg/kg), as described in 2.3. Total deoxyribonucleic acid (DNA) was extracted from the colonic contents using an Omega Soil DNA Kit (M5635-02; Omega Bio-Tek, Norcross, GA, USA), and DNA quality was assessed *via* agarose gel electrophoresis. The bacterial 16S ribosomal ribonucleic acid (rRNA) V3–V4 region was amplified using forward (ACTCCTACGGGGAGGCAGCA) and reverse (GGACTACHVGGGTWTCTAAT) primers. Polymerase chain reaction (PCR) amplification products were quantified using a Quant-iT PicoGreen dsDNA Assay Kit (Invitrogen, Carlsbad, CA, USA). Equal amounts of amplified products were pooled and subjected to paired-end 2 × 250 base pair (bp) sequencing on an Illumina NovaSeq platform using a NovaSeq 6000 SP reagent kit (500 cycles).

Raw sequences were initially processed using the ‘demux’ plugin, and primer removal was subsequently performed using the ‘cutadapt’ plugin. Data integration was conducted using the DADA2 package, and sequences were identified by referencing the SILVA taxonomy release 138.1 database.

### 2.12. Non-targeted metabolomics analysis

To begin, incorporate 50 milligrams of fecal specimen into 500 microliters of pre-chilled methanol that contains 5 parts per million of 2-chlorophenylalanine. Subsequently, homogenize the mixture at a frequency of 55 hertz for 60 s, repeating this process one additional time. Following this, subject the sample to sonication for duration of 10 min. Then, transfer the preparation to a −20 °C freezer for 30 min, followed by centrifugation at 4 °C and 12,000 rpm for 10 min. Finally, carefully collect the resulting supernatant. The liquid chromatography/mass spectrometry (LC/MS) setup featured an ACQUITY UPLCHSS T3 column paired with a Thermo Orbitrap Exploris 120 mass spectrometer. We utilized Compound Discoverer^™^ 3.3 to handle peak extraction, alignment, and correction, while metabolite classification and identification were carried out using a combination of our in-house library, the mzCloud online database, LIPID MAPS, HMDB, MoNA, and the NIST_2020_MSMS spectral library.

### 2.13. Data processing and analysis

This study utilized ImageJ for analyzing protein bands and fluorescence images, performed one-way ANOVA using IBM SPSS Statistics 27.0, and generated graphs with GraphPad Prism 8.0. The data were tested for normal distribution using the Shapiro-Wilk test. The data with normal distribution were analyzed for intergroup differences using one-way ANOVA or Welch’s test followed by LSD or Tamhane’s T2 test. Non-normal distribution data were analyzed by Kruskal-Wallis one-way ANOVA. Compared with ‘#’, *P*-value < 0.05 indicates significant differences. Sequence data analysis employed QIIME2 and the R package (v3.2.0) to calculate α-diversity metrics at the ASV level. β-diversity analysis utilized LEfSe (Linear Effect Size of discriminant analysis) to detect intergroup differences in abundance-based taxonomic units. Metabolome data underwent orthogonal partial least squares discriminant analysis (OPLS-DA) and differential metabolite analysis using R software. Differential metabolites underwent KEGG enrichment analysis *via* clusterProfiler (v4.6.0).

## 3. Results

### 3.1. FIS attenuates LPS-induced inflammatory response in MAC-T cells

**Figure 1.**
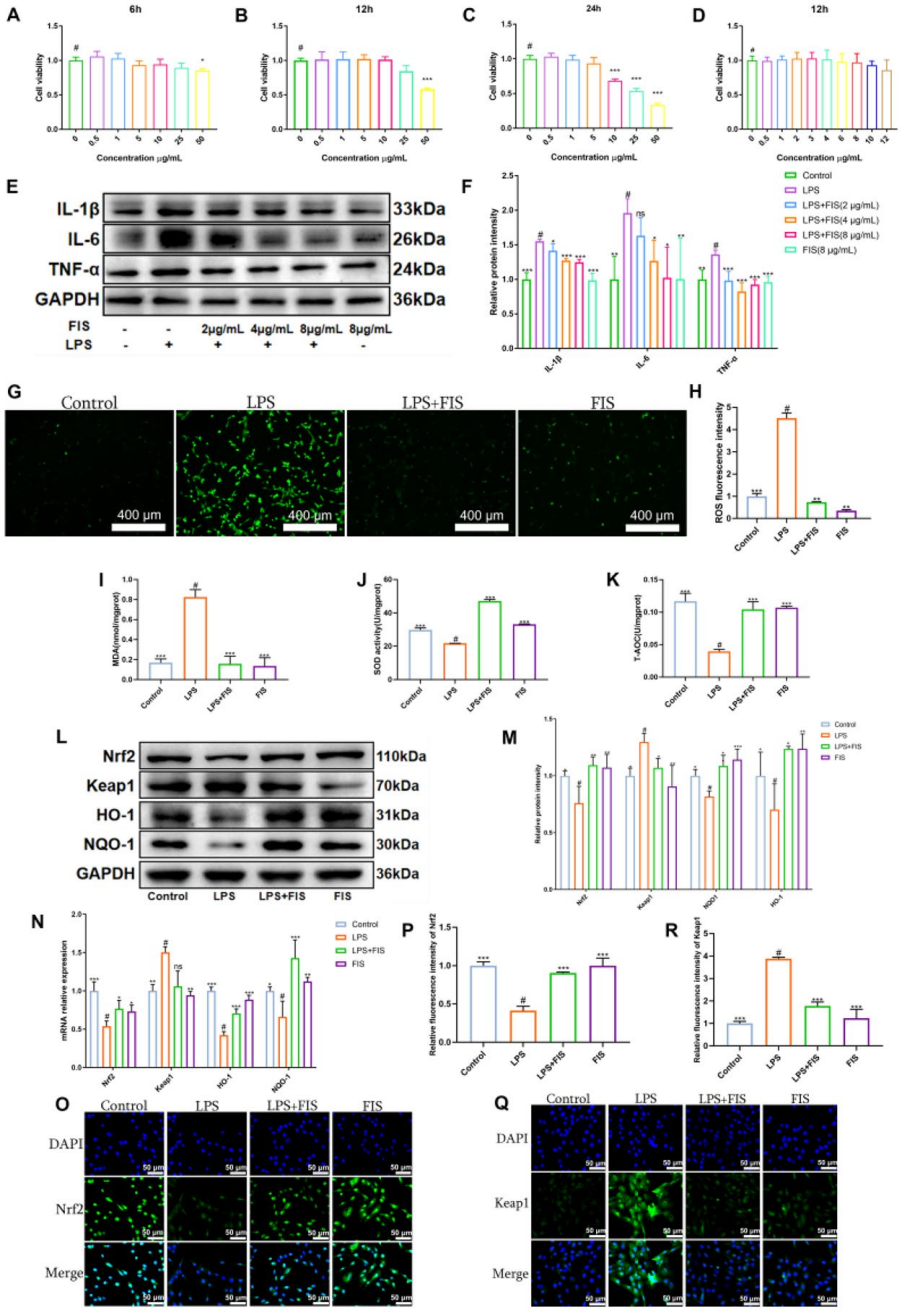
FIS attenuates LPS-induced inflammatory response in MAC-T cells. (A-D) MAC-T cells viability following FIS treatment. (E,F) Western blot bands and quantification histograms of IL-1β, TNF-α, and IL-6 protein expression. (G,H) ROS fluorescence images and quantification histograms. (I) MDA content. (J) SOD content. (K) T-AOC levels. (L,M) Western blot bands and quantification histograms of Nrf2, Keap1, HO-1, and NQO-1 protein expression. (N) mRNA levels of *Nrf2, Keap1, NQO-1*, and *HO-1*. (O,P) Immunofluorescence intensity and quantitative analysis of Nrf2. (Q,R) Immunofluorescence intensity and quantitative analysis of Keap1. Data are presented as the mean ± SD (*n* = 3 per group), *compared to ‘#’, ‘#’ refers to the LPS group, *p < 0.05, **p < 0.01, ***p < 0.001; ns: not significant.

We initially screened the effective concentration of FIS by assessing its impact on MAC-T cell viability. The cells were treated with 0–50 μg/mL FIS for 6, 12, and 24 h. We observed that 10 μg/mL FIS, administered for 12 h, did not significantly alter cell viability (Figure 1A–C). Subsequently, cells were treated with 0–12 μg/mL FIS for 12 h (Figure 1D), and concentrations of 2, 4, and 8 μg/mL were selected for preliminary experiments. Pretreatment of cells with FIS suppressed the protein expression of IL-1β, IL-6, and TNF-α (Figure 1E,F), with 4 μg/mL demonstrating a marked inhibitory effect. Consequently, we used 4 μg/mL FIS in the subsequent experiments.

### 3.2. Effects of FIS on oxidative stress in LPS-induced MAC-T cells

We further investigated whether the anti-inflammatory effects of FIS were associated with oxidative stress. We examined the levels of ROS, MDA, SOD, and T-AOC, as well as the expression of Keap1, Nrf2, NQO-1, and HO-1 proteins and corresponding mRNA levels, along with immunofluorescence of key proteins. Compared to the Control group, ROS had a significant increase in the LPS group, while LPS+FIS group significantly reduced ROS levels (Figure 1G,H). MDA, SOD, and T-AOC levels in the cells (Figure 1I–K) showed that the LPS group had significantly elevated MDA content, reduced SOD activity, and a significant decrease in T-AOC, while the FIS treatment group had significantly decreased MDA levels, increased SOD content, and elevated T-AOC. Western blot analysis (Figure 1L,M) revealed that FIS enhanced the expression of Nrf2, HO-1, and NQO-1 while reducing the expression of Keap1. The mRNA levels were consistent with the above protein results (Figure 1N), indicating that FIS regulates oxidative stress balance by enhancing the Nrf2, HO-1, and NQO-1 pathways through increased expression at the gene level. The cell immunofluorescence of Nrf2 (Figure 1O,P) and Keap1 (Figure 1Q,R) results further confirmed this.

### 3.3. Effects of FIS on ferroptosis in LPS-induced MAC-T cells

**Figure 2.**
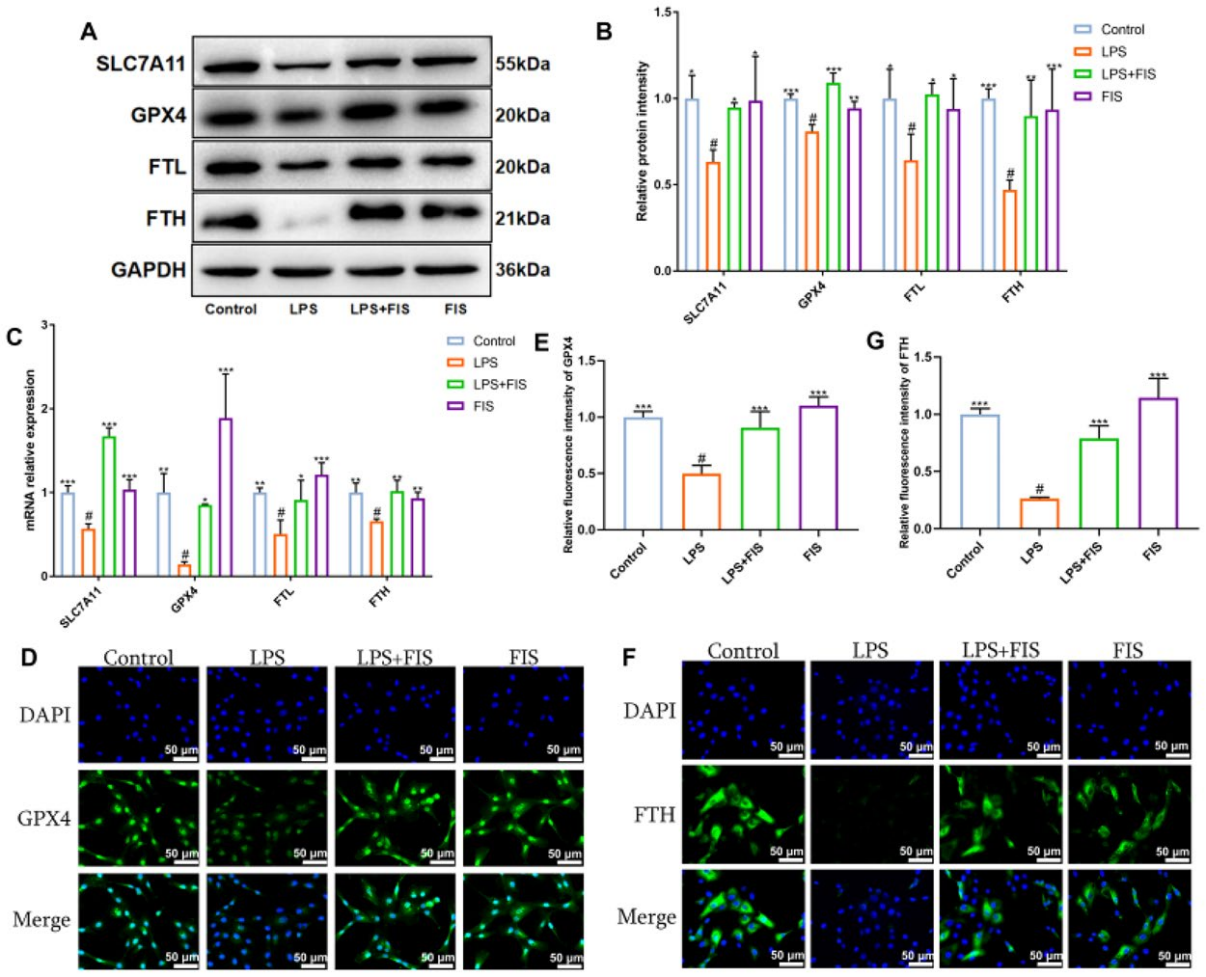
Effects of FIS on ferroptosis in LPS-induced MAC-T cells. (A,B) Western blot analysis and quantification of SLC7A11, GPX4, FTL, and FTH protein expression. (C) The expression of SLC7A11, GPX4, FTL, and FTH mRNA levels. (D,E) Immunofluorescence intensity and quantification of GPX4. (F,G) Immunofluorescence intensity and quantification of FTH. Data are presented as the mean ± SD (*n* = 3 per group), *compared to ‘#’, ‘#’ refers to the LPS group, *p < 0.05, **p < 0.01, ***p < 0.001; ns: not significant.

We investigated the expression of SLC7A11, GPX4, FTL, and FTH proteins and mRNA, as well as the cellular immunofluorescence of GPX4 and FTH. Initially, Western blot analysis revealed that LPS stimulation significantly suppressed the expression of SLC7A11, GPX4, FTL, and FTH proteins. Conversely, the LPS+FIS group markedly enhanced the expression of SLC7A11, GPX4, FTL, and FTH proteins (Figure 2A,B). QPCR results showed a significant decrease in GPX4 mRNA levels in the LPS group, along with varying degrees of reduction in SLC7A11, FTH, and FTL levels. The LPS+FIS group significantly upregulated SLC7A11 expression, and GPX4, FTH, and FTL levels (Figure 2C). Cellular immunofluorescence results also indicated a marked reduction in GPX4 (Figure 2D,E) and FTH (Figure 2F,G) expression in the LPS group, whereas the LPS+FIS group exhibited a significant increase. These findings collectively suggest that FIS can alleviate ferroptosis in LPS-induced MAC-T cells by regulating the SLC7A11/GPX4 signaling pathway.

### 3.4. Effects of FIS on LPS-induced mammary gland damage and the blood-milk barrier in mice

**Figure 3.**
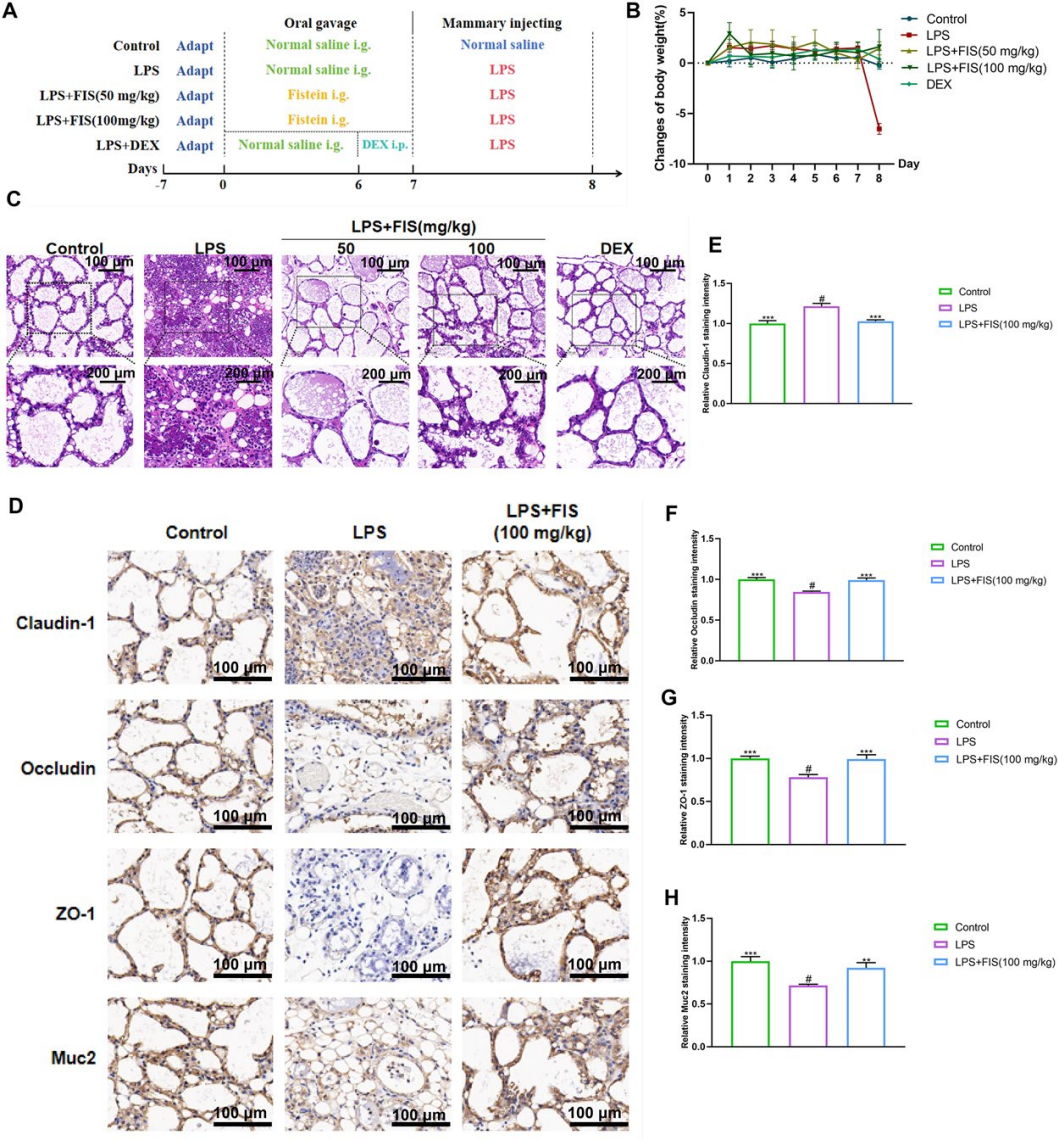
Effects of FIS on LPS-induced mammary gland damage and the blood-milk barrier in mice. (A) Experimental design flowchart. (B) Body weight changes in mice, presented as a line graph, (*n* = 6). (C) HE staining of mammary gland tissue from mice. (D-H) Immunohistochemical staining and quantification of Claudin-1, occludin, ZO-1, and Muc2 protein expression.Data are presented as the mean ± SD (*n* = 3 per group), *compared to ‘#’, ‘#’ refers to the LPS group, *p < 0.05, **p < 0.01, ***p < 0.001; ns: not significant.

We subsequently investigated the protective effects of FIS on mammary gland tissue, which was verified by recording daily body weight changes in mice, tissue HE staining and immunohistochemistry. As shown in Figure 3(A), the experimental animal grouping and operational procedures are presented. Following this, by recording the changes in mouse body weight (Figure 3B), it was found that the body weight of the mice was basically stable before LPS modeling. After modeling, the body weight of LPS group had a significant decrease compared to the other groups, indicating that LPS has a strong stimulatory effect on mice. HE staining revealed that the structure of the mammary gland tissue in the LPS group was disrupted, with a large number of inflammatory cell infiltrations. In contrast, the FIS group showed a significant reduction in inflammatory cells (Figure 3C). Immunohistochemical results showed that the expression of Claudin-1 was significantly upregulated in the mammary gland tissue of the LPS group, while the expression of Occludin, ZO-1, and Muc2 were significantly downregulated. Conversely, the protein expression of Claudin-1 was downregulated, while the proteins of Occludin, ZO-1, and Muc2 were significantly upregulated in the LPS+FIS (100 mg/kg) group (Figure 3D–H). These results suggest that FIS can protect against LPS-stimulated mammary gland damage by reducing inflammatory cell infiltration and simultaneously restoring the blood-milk barrier.

### 3.5. Effects of FIS on oxidative stress in murine mammary tissue

**Figure 4.**
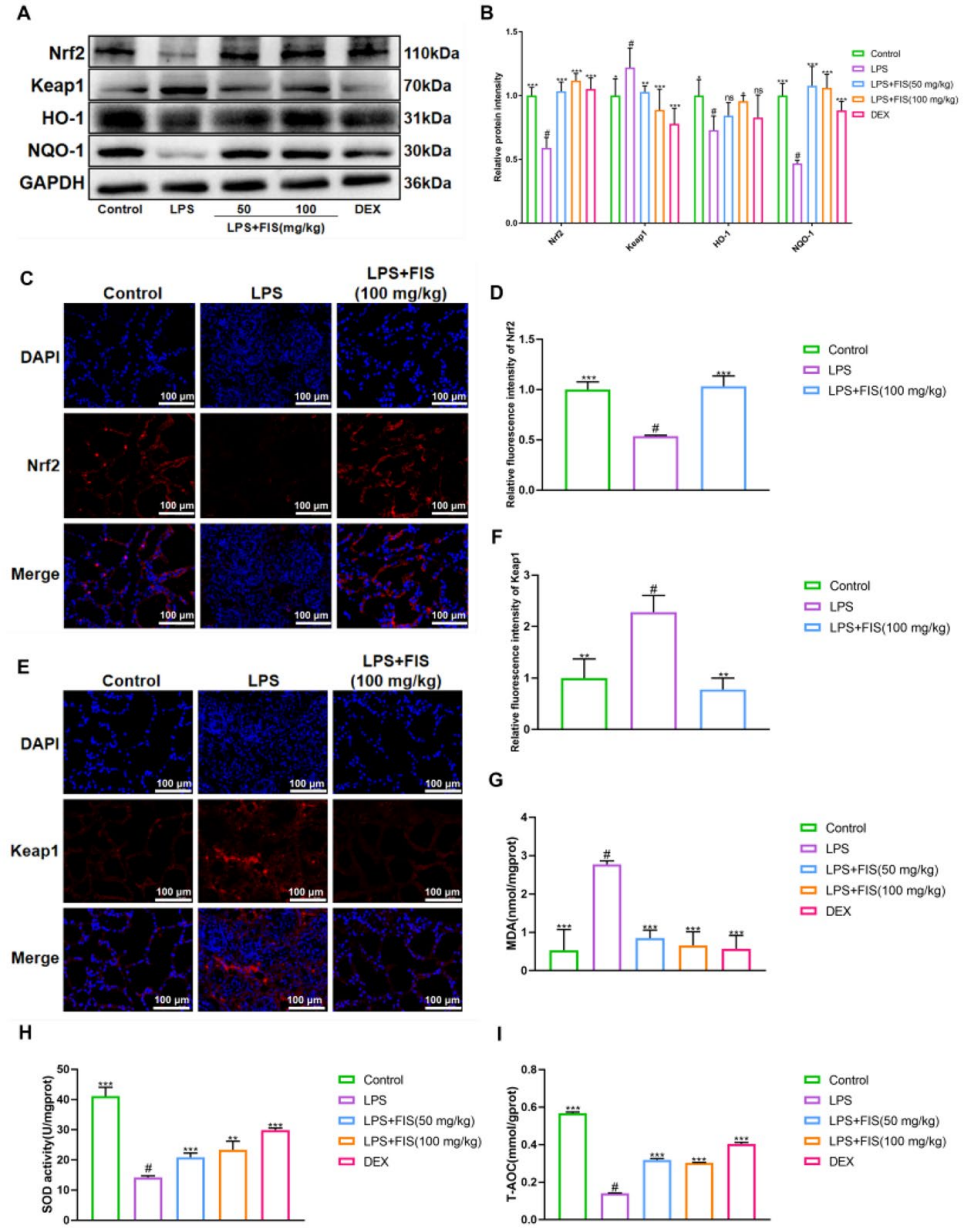
Effects of FIS on oxidative stress in murine mammary tissue. (A,B) Western blot bands and quantification of Nrf2, Keap1, NQO-1, and HO-1 protein expression in mammary gland tissue from mice. (C,D) Immunofluorescence images and quantification of Nrf2 protein in mammary gland tissue from mice. (E,F) Immunofluorescence images and quantification of Keap1 protein in mammary gland tissue from mice. (G-I) MDA, SOD, and T-AOC levels in mammary gland tissue from mice. Data are presented as the mean ± SD (*n* = 3 per group), *compared to ‘#’, ‘#’ refers to the LPS group, *p < 0.05, **p < 0.01, ***p < 0.001; ns: not significant.

To investigate whether FIS exerts therapeutic effects on mastitis *via* oxidative stress modulation, we assessed the expression of oxidative stress-related proteins, tissue immunofluorescence, and the levels of MDA, SOD, T-AOC. Western blot analysis (Figure 4A,B) showed a significant elevation of Keap1 protein and downregulation of Nrf2, HO-1, and NQO-1 protein expression in the LPS group. The FIS group exhibited the opposite trend, restoring the expression of Nrf2, NQO-1, and HO-1 proteins. Tissue immunofluorescence, as shown in Figure 4(C–F), with increased Nrf2 content and decreased Keap1 content in the tissue compared to the LPS group. The results of the kit-based assays showed a increase in MDA levels and a significant decrease in SOD and T-AOC levels in the LPS group. In contrast, the FIS-treated mice showed decreased MDA levels and increased SOD and T-AOC levels in their mammary tissue (Figure 4G–I). These findings suggest that FIS protects mammary tissue from inflammatory damage induced by oxidative stress by modulating the Keap-Nrf2 pathway and upregulating the levels of antioxidant enzymes in the tissue.

### 3.6. Effect of FIS on ferroptosis in murine mammary tissue

**Figure 5.**
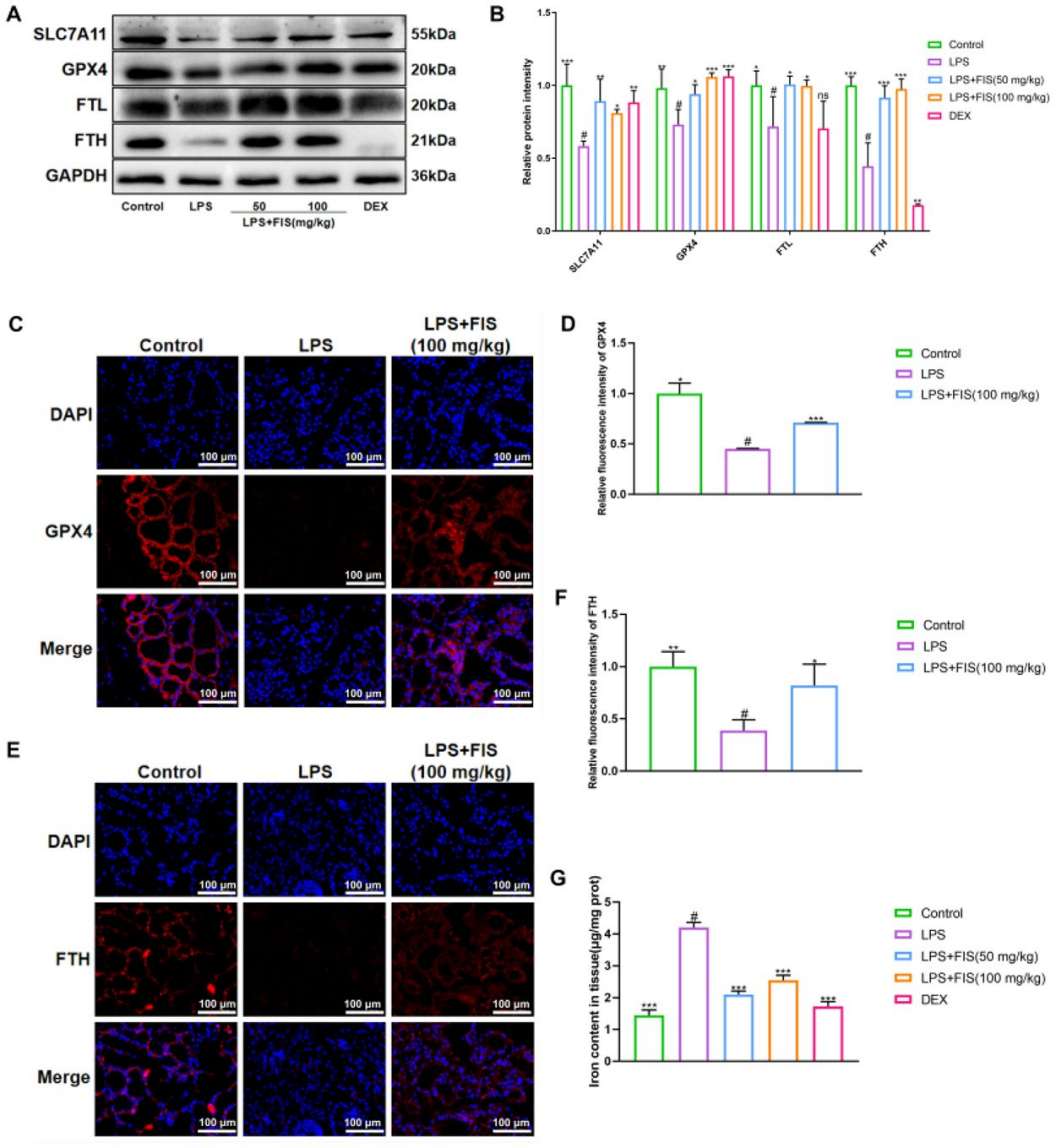
Effect of FIS on ferroptosis in murine mammary tissue. (A,B) Western blotting bands and quantification of SLC7A11, GPX4, FTL, and FTH protein expression in mammary gland tissue from mice. (C,D) Immunofluorescence images and quantification of GPX4 protein. (E,F) Immunofluorescence images and quantification of FTH protein. (G) Total iron content in mammary gland tissue from mice. Data are presented as the mean ± SD (*n* = 3 per group), *compared to ‘#’, ‘#’ refers to the LPS group, *p < 0.05, **p < 0.01, ***p < 0.001; ns: not significant.

Subsequently, we investigated the role of FIS in ferroptosis, which was validated through the expression of SLC7A11, GPX4, FTL and FTH proteins, tissue immunofluorescence, and the total iron content of the tissue. Western blot analysis revealed that the expressions of SLC7A11, GPX4, FTH, and FTL were significantly decreased in the LPS group. Conversely, the FIS treatment group exhibited increased protein expression of SLC7A11, GPX4, FTL, and FTH (Figure 5A,B). Tissue immunofluorescence results indicated a reduction in GPX4 and FTH fluorescence intensity in the LPS group, whereas the FIS group showed a marked enhancement (Figure 5C–F), consistent with the protein expression findings. Total iron content in the tissue was measured using a kit (Figure 5G). Compared with the control group, the total iron content in mammary tissue was significantly increased in the LPS group, while it was significantly decreased in the FIS-treated group. These results indicate that the FIS treatment group reduces Fe^2+^ to Fe³^+^ within tissues by increasing FTH and FTL expression, while simultaneously promoting cysteine transport. This ultimately enhances GPX4 expression and inhibits the generation of lipid peroxides.

### 3.7. Effects of FIS on intestinal microbiota diversity and composition in mice with LPS-induced mastitis

**Figure 6.**
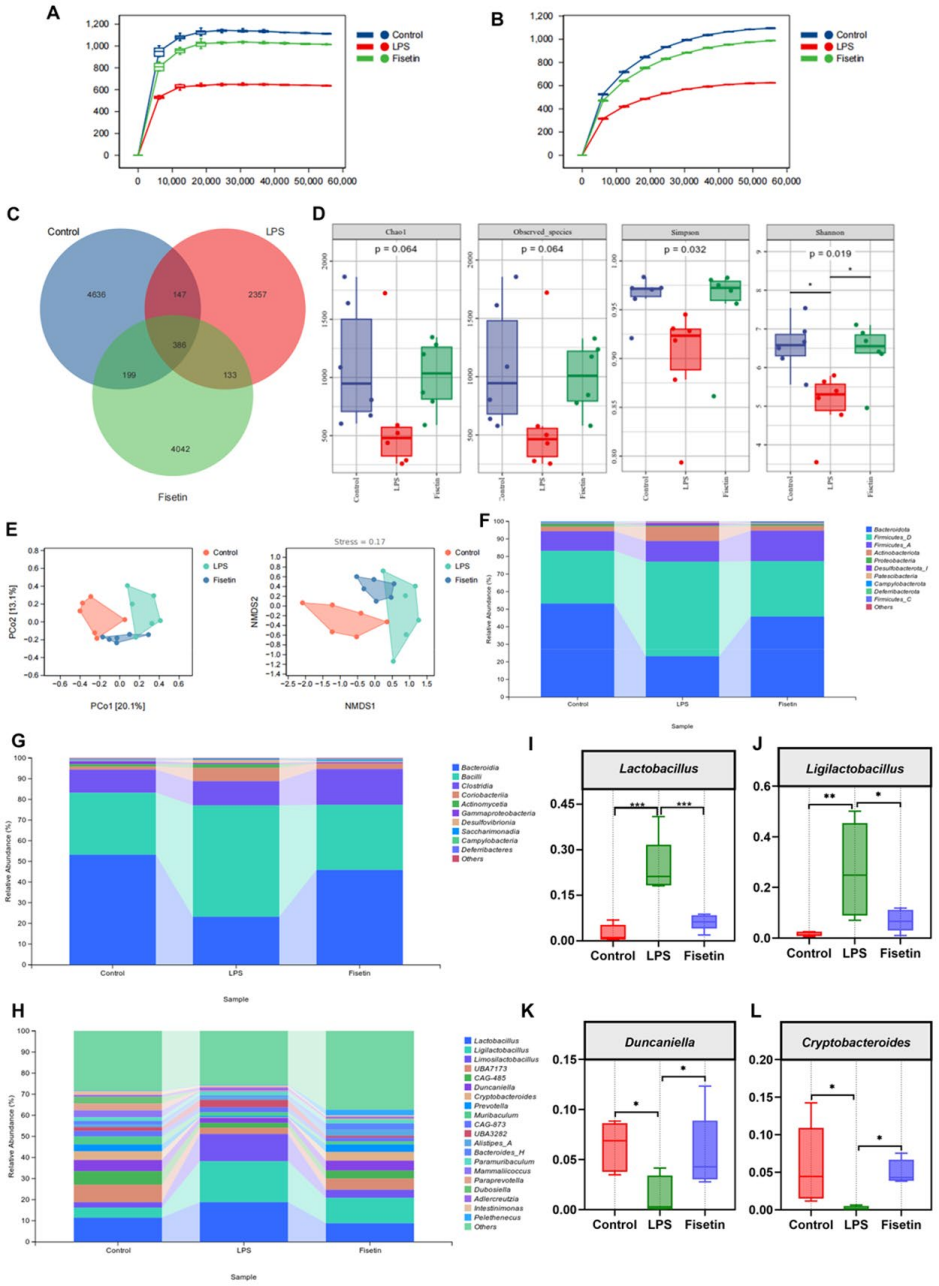
Effects of FIS on intestinal microbiota diversity and composition in mice with LPS-induced mastitis. (A,B) Analysis of Chao1 and observed-species indices of the gut microbiota. (C) OTU clustering of the gut microbiota across groups. (D) Chao1, observed-species, simpson, and shannon indices of the gut microbiota.(E) PCoA analysis and NMDS analysis. (F) Relative abundance of gut microbiota at the phylum level. (G) Relative abundance of gut microbiota at the family level. (H) Relative abundance of gut microbiota at the genus level. (I) Relative abundance of *lactobacillus*. (J) Relative abundance of *ligilactobacillus*. (K) Relative abundance of *duncaniella*. (L) Relative abundance of *cryptobacteroides*. Data are presented as the mean ± SD (*n* = 6 per group), *compared to the LPS group, *p < 0.05, **p < 0.01, ***p < 0.001.

As shown in Figure 6(A,B), the sparse curve indicates that the current sequencing depth has reached a plateau, meaning that the majority of gut communities across all samples have been sufficiently captured. OTU analysis revealed 4636, 2357, and 4042 unique OTUs in the Control, LPS, and Fisetin groups, respectively (Figure 6C). This indicates significantly reduced gut microbial diversity in the LPS group, with distinct microbial community structures compared to the Control and Fisetin groups. As shown in Figure 6(D), α-diversity analysis revealed that LPS treatment induced pronounced alterations in microbial abundance and diversity. Compared to the Control and Fisetin groups, the Shannon index in the LPS group decreased significantly. Although other indices did not reach statistical significance, they also decreased. β-diversity analysis (Figure 6E,F) revealed a distinct separation in gut microbial structure between the LPS and Control groups. However, FIS group markedly restored the composition of gut microbial structure. At the phylum level, LPS induction reduced Bacteroidetes abundance while increasing Firmicutes-D abundance (Figure 6G). At the family level, the dominant families were Bacteroidia and Bacilli. LPS significantly downregulated Bacteroidia abundance while upregulating Bacilli abundance, whereas the Fisetin and Control groups showed the opposite pattern (Figure 6H). At the genus level, compared to the Control and Fisetin groups, LPS group induction significantly upregulated *Lactobacillus* and *Ligilactobacillus* abundance while downregulating *Duncaniella* and *Cryptobacteroides* abundance (Figure 6 I-L).

**Figure 7.**
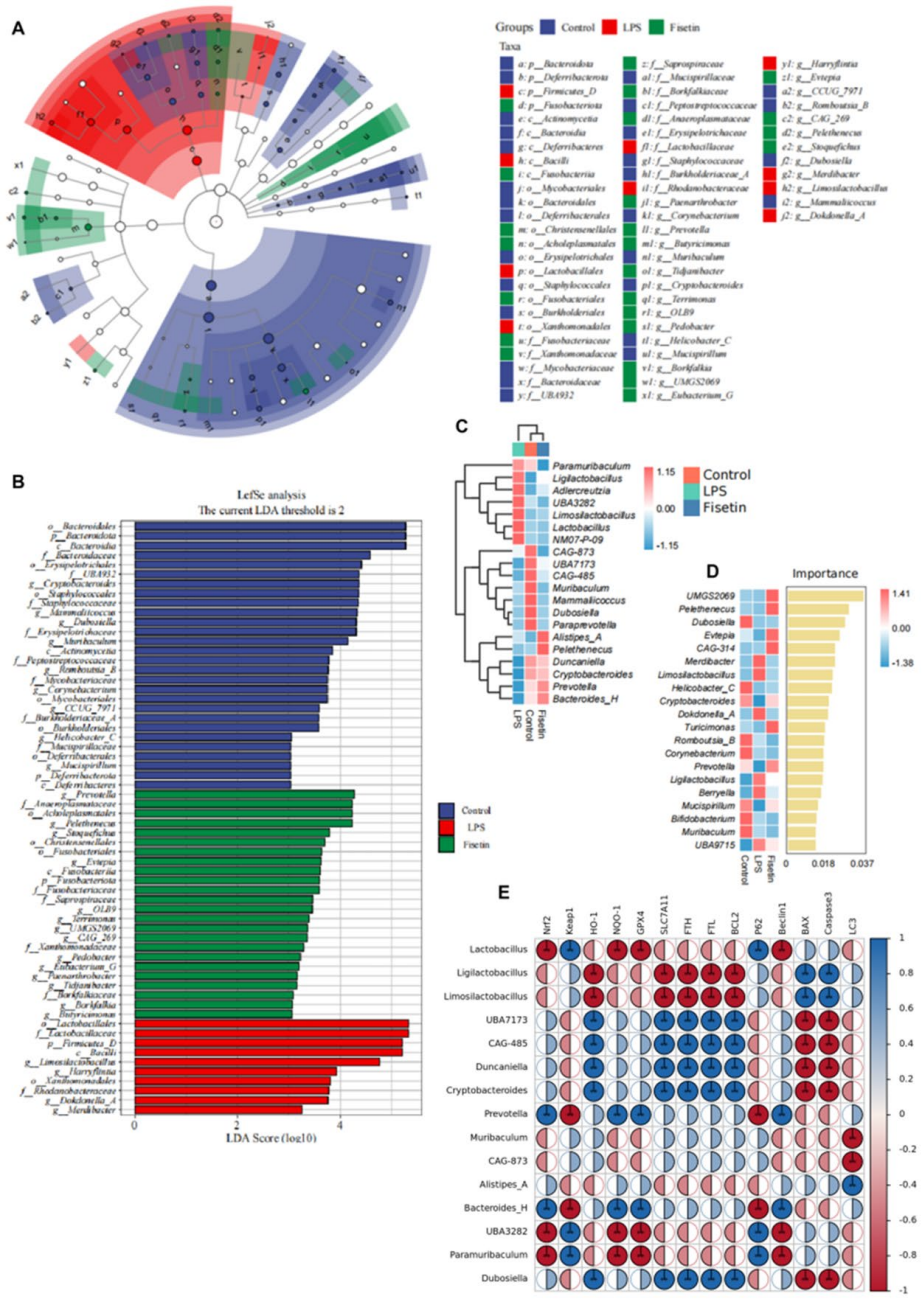
Effects of FIS on intestinal microbiota diversity and composition in mice with LPS-induced mastitis. (A,B) LEfSe analysis of gut microbiota, with circles representing taxonomic levels from phylum to species, and the size of each point proportional to its abundance. (C) Heatmap analysis of gut microbiota composition. (D) Random forest analysis of gut microbiota. (E) Spearman correlation analysis of the top 15 genera identified by random Forest with protein expression in 14 mammary gland tissues. *p < 0.05, **p < 0.01, ***p < 0.001, *n* = 6.

Linear discriminant analysis effect size (LEfse) analysis (LDA score > 3) revealed 62 distinct clusters across the Control, LPS, and Fisetin groups. The dominant bacterial communities differed significantly among groups. Lactobacillales, Lactobacillaceae, and Firmicutes_D were enriched in the LPS group, while *Prevotella*, _Anaeroplasmataceae, and Acholeplasmatales were enriched in the Fisetin group (Figure 7A,B). Species heatmap composition and random forest analysis revealed distinct dominant bacterial communities between the LPS group and the Control and Fisetin groups. *Limosilactobacillus*, *Ligilactobacillus*, and *Lactobacillus* were significantly enriched in the LPS group, while *Pelethenecus*, *Duncaniella, Cryptobacteroides*, and *Prevotella* were significantly enriched in the Fisetin group (Figure 7C,D). Spearman correlation analysis was performed between the top 15 most significant bacterial genera at the genus level and proteins associated with ferroptosis and oxidative stress (Figure 7E). *Lactobacillus acidophilus, Lactobacillus reuteri,* and *Lactobacillus* were enriched in the LPS group, positively correlated with Keap1, and negatively correlated with Nrf2, NQO1, GPX4, HO-1, SLC7A11, and FTH. Conversely, *Duncaniella, Cryptobacteroides*, and *Prevotella* exhibited negative correlations with Nrf2, NQO1, GPX4, and HO-1, while positive correlations with FTH. FTH, and FTL. Conversely, the genera *Duncaniella, Cryptobacteroides*, and *Prevotella*, significantly enriched in the Fisetin group, showed positive correlations with Nrf2, NQO1, GPX4, HO-1, SLC7A11, FTH, and FTL, and negative correlations with Keap1.

### 3.8. Effect of FIS on metabolite profiles in mice with LPS-induced mastitis

**Figure 8.**
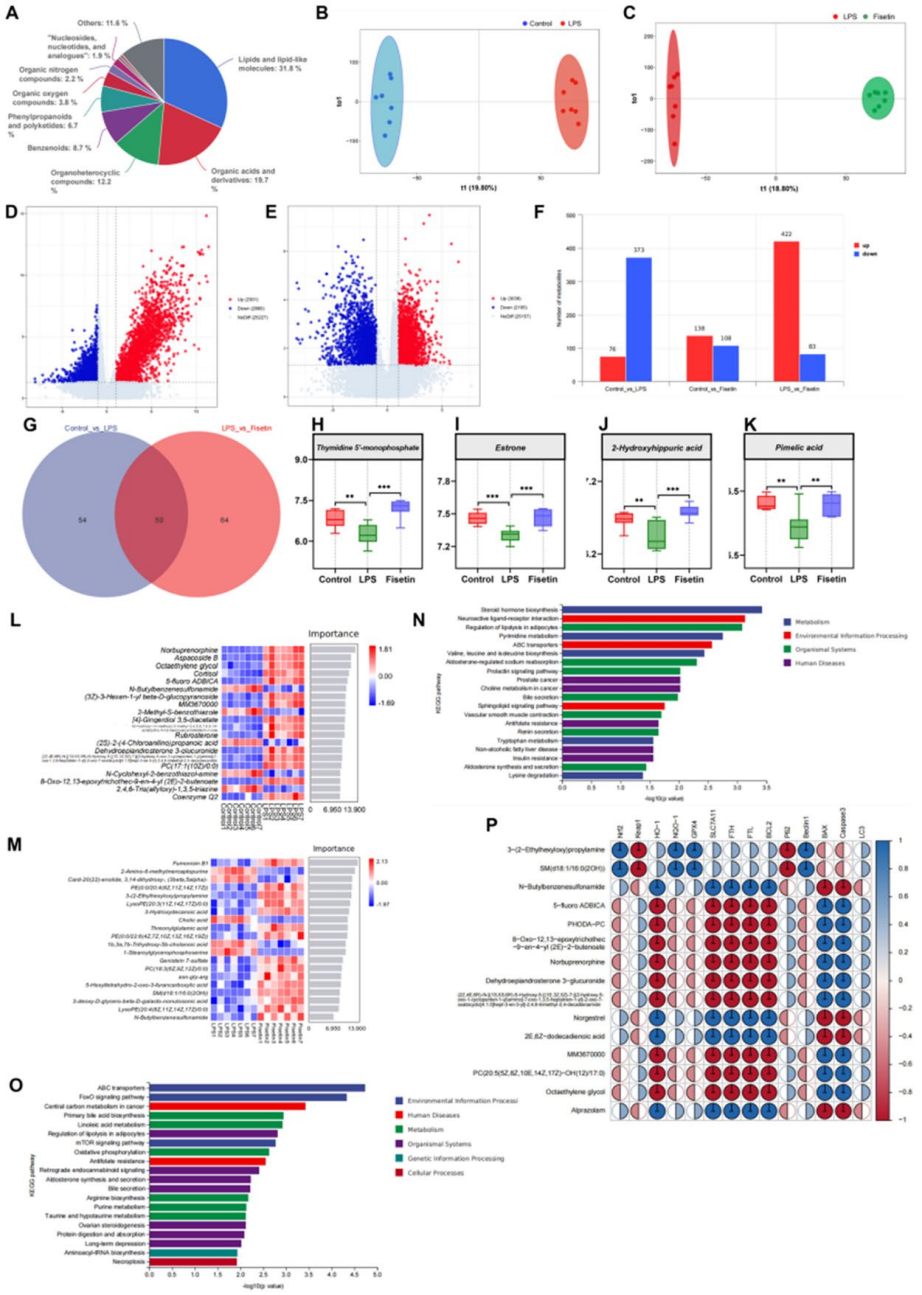
Effect of FIS on metabolite products in mice with LPS-induced mastitis. (A) Top ten metabolite species in the first classification. (B,C) OPLS-DA analysis of gut metabolites across groups. (D) Volcano plot analysis of metabolites in the control and LPS groups. (E) Volcano plot analysis of metabolites in the LPS and fisetin groups. (F) Number of upregulated and downregulated metabolites in pairwise comparisons. (G) Relative abundance of thymidine 5′-monophosphate. (H) Relative abundance of estrone. (I) Relative abundance of 2-Hydroxyhippuric acid. (J) Relative abundance of pimelic acid. (K) Relative abundance of ligilactobacillus in each group. (L) Heatmap analysis of metabolite composition in the control and LPS groups. (M) Heatmap analysis of metabolite composition in the LPS and fisetin groups. (N) Random forest analysis of metabolites in the control and LPS groups. (O) Random forest analysis of metabolites in the LPS and fisetin groups. (P) Spearman correlation analysis of 15 common differential metabolites across the three groups with protein expression in 14 mammary gland tissues. Data are presented as the mean ± SD (*n* = 6 per group), *compared to the LPS group, * *p* < 0.05, **p < 0.01, ***p < 0.001.

Non-targeted metabolomics analysis of colonic contents revealed that the predominant metabolites categorized under the first-level classification included lipids and lipid-like molecules (31.8%), organic acids and derivatives (19.7%), and organoheterocyclic compounds (12.2%) (Figure 8A). Orthogonal projections to latent structures discriminant analysis (OPLS-DA) demonstrated clear separation of metabolite profiles among the groups (Figure 8B,C), indicating distinct metabolic changes. Differential analysis of all metabolites showed that the LPS group exhibited 2,301 upregulated and 2,860 downregulated metabolites compared to the Control group. Conversely, the Fisetin group exhibited 3,036 upregulated and 2,195 downregulated metabolites compared with the LPS group (Figure 8D,E). Differential metabolites were screened using OPLS-DA with a variable importance in projection (VIP) value >1 and *p* < 0.05. Compared with the Control group, the LPS group presented 76 upregulated and 373 downregulated metabolites. Relative to the LPS group, the Fisetin group showed 422 upregulated and 83 downregulated metabolites (Figure 8F). A Venn diagram analysis identified 50 key metabolites among the differential metabolites between the Control vs. LPS and LPS vs. Fisetin groups (Figure 8G). Among these, the levels of four key differential metabolites, thymidine 5′-monophosphate, estrone, 2-hydroxyhippuric acid, and pimelic acid, were significantly decreased in the LPS group compared to the Control and Fisetin groups (Figure 8H–K). Heatmap visualization of the top 20 most important metabolites revealed that norbuprenorphine, aspacoside B, and octaethylene glycol were the primary differential metabolites between the Control and LPS groups, whereas fumonisin B1, 2-amino-6-methylmercaptopurine, and PE(0:0/20:4[8Z,11Z,14Z,17Z]) were prominent in LPS and Fisetin groups (Figure 8L,M). KEGG pathway enrichment analysis of differential metabolites (Figure 8N,O) revealed that the top 20 metabolic pathways annotated by differentially expressed metabolites between the LPS and Control groups were mainly enriched in ‘Steroid hormone biosynthesis,’ ‘Neuroactive ligand-receptor interaction,’ and ‘Regulation of lipolysis in adipocytes.’ The LPS and Fisetin groups were mainly enriched in ‘ABC transporters,’ ‘FoxO signaling pathway,’ and ‘Central carbon metabolism in cancer.’ Spearman correlation analysis of the top 15 differential metabolites and key pathway-related proteins revealed that 3-(2-ethylhexyloxy)propylamine and SM(d18:1/16:0(20H)) were positively correlated with Nrf2, NQO-1, and GPX4 (*p* < 0.01) and negatively correlated with Keap1 (*p* < 0.01). Additionally, N-butylbenzenesulfonamide, norgestrel, 2E,6Z-dodecadienoic acid, and alprazolam were positively correlated with HO-1, SLC7A11, FTH, and FTL (*p* < 0.01), whereas the remaining metabolites, including 5-fluoro ADBICA, PHODA-PC, and norbuprenorphine, were negatively correlated with HO-1, SLC7A11, FTH, and FTL (*p* < 0.01) (Figure 8P). Among the top three key metabolites, 3-(2-ethylhexyloxy)propylamine and SM(d18:1/16:0(20H)) levels increased in the Fisetin group and decreased in the LPS group, whereas N-Butylbenzenesulfonamide exhibited the opposite pattern. Thus, LPS-induced mastitis disrupted the metabolic profile by upregulating metabolites associated with Keap1. Fisetin treatment reversed the change, mitigating the LPS-induced disruption of the metabolic profile.

## 4. Discussion

The administration of antibiotics remains the primary therapeutic intervention for bovine mastitis; however, the escalating misuse of antibiotics and the concomitant emergence of antibiotic-resistant bacteria threaten environmental and human health worldwide. Consequently, herbal medicines, which are characterized by their low toxicity and minimal propensity for resistance development, have been considered to be promising alternatives (Qadri et al. 2022; Suganya et al. 2022). The results revealed that FIS effectively suppressed the expression of pro-inflammatory cytokines, including interleukin 6 (IL-6), interleukin 1 beta (IL-1β), and tumor necrosis factor alpha (TNF-α) in MAC-T cells, thereby mitigating the pathogenesis of mastitis. The results are consistent with those of other studies that demonstrated the potent anti-inflammatory properties of FIS were mediated by inhibiting the nuclear factor kappa B (NF-κB) pathway (Liu et al. 2025), the amelioration of LPS-induced endometritis *via* the suppression of the TLR4 pathway, and the reduction of inflammatory marker expression (TNF-α, IL-1β, and IL-6), thereby alleviating renal inflammation in rats with diarrhea (Koriem and Farouk 2023).

The Keap1/Nrf2 pathway plays an importent role in modulating oxidative stress responses. The results revealed that FIS enhanced the transcription and expression of Nrf2, NQO-1, and HO-1, increased SOD levels, and elevated the total antioxidant capacity, while simultaneously inhibiting the transcription and expression of Keap1, thereby limiting the LPS-induced generation of ROS. The *in vivo* findings corroborated the *in vitro* results. Previous studies have reported that lentinan, a polysaccharide, exerts its anti-inflammatory and antioxidant effects by modulating the Nrf2 pathway to counteract LPS-induced damage in bovine mammary epithelial cells (BMECs) (Meng et al. 2022) and that salvianolic acid attenuates mastitis-induced damage through the regulation of the Nrf2/mitogen-activated protein kinase (MAPK) pathway (Yang et al. 2025). These finds underscore the critical role of Nrf2 in these processes and highlight the potential utility of targeting Nrf2 to modulate HO-1 activation and reduce inflammatory responses in the therapeutic management of mastitis. Numerous studies have established the robust antioxidant activity of FIS, mediated through the activation of Nrf2/NQO-1/HO-1 signaling pathways (Park et al. 2023; Qian et al. 2023; Liu and Lu 2024); the present findings are consistent with the results of those studies and effectively demonstrate that FIS inhibits mammary gland inflammation and oxidative stress damage *via* the activation of Nrf2.

Ferroptosis, a form of iron-dependent cell death, is induced by the intracellular catalytic activity of labile ferrous iron, which first promotes lipid peroxidation. Previous studies have demonstrated that E. coli can trigger ferroptosis in BMECs (Zhuang et al. 2024) and that ferroptosis also occurs in LPS-induced mammary gland injury (Sun et al. 2025). In this study, LPS not only induced cellular inflammatory responses, it also stimulated ferroptosis, suggesting a close association may exist between the onset of bovine mastitis and ferroptosis. LPS significantly suppressed the protein expression levels of SLC7A11, GPX4, FTL, and FTH, and FIS mitigated both ferroptosis and cellular inflammatory responses by promoting the expression of these proteins. The *in vivo* experiments further confirmed that FIS exerted anti-inflammatory effects through the inhibition of ferroptosis, thereby effectively alleviating mastitis in mice. Previous studies have demonstrated that FIS can inhibit ferroptosis by suppressing ACSL4, thereby ameliorating ferroptosis in murine models of fibrotic nephropathy (Wang et al. 2024). Additionally, FIS has been shown to inhibit ferroptosis by activating the sirtuin 1 (SIRT1)/Nrf2 pathway (Li et al. 2021), consistent with the present findings.

The blood-milk barrier (BMB) is a crucial barrier to protect the health of mammary tissue. The core barrier structure is composed of tight junction proteins. Our research revealed that after LPS stimulation of mice, Claudin-1 protein expression increased in mammary tissue, while Occludin, ZO-1, and Muc2 protein expression decreased, indicating that LPS destroyed BMB. After FIS treatment, abnormal protein expression was restored, suggesting that FIS protects mammary tissue by restoring the BMB. Ken Kobayashi’s research found that Claudin-1 protein expression increased significantly 12 h after LPS stimulation, which is potentially related to the TLR4 signaling pathway activated by LPS (Kobayashi et al. 2013). This finding is consistent with ours.

Extensive research has established a link between the gut microbiota, its metabolites, and the development of mastitis. For example, fecal microbiota transplantation from mastitis-affected cows into germ-free mice has been shown to induce mastitis-like symptoms in the recipient animals (Ma et al. 2018). Furthermore, mice with mastitis often exhibit a reduction in gut microbiota richness and a decrease in the levels of beneficial metabolites, and supplementation of these metabolites can improve mastitis symptoms (Wang et al. 2024). The present study revealed significant alterations in both the structure of the gut microbiota and the composition of metabolites in mice with LPS-induced mastitis compared to that of mice in the control group. More specifically, there was a downregulation of the Bacteroidetes phylum following the establishment of LPS-induced mastitis. This reduction in *Bacteroidetes* led to the decreased production of SCFAs, such as butyrate, which, in turn, reduced the production of pro-inflammatory cytokines and nitric oxide and inhibited the expression of ZO-1, thereby compromising the blood–milk barrier (Gerunova et al. 2024). In the Fisetin group, there was an upregulation of the Bacteroidetes phylum and an increase in the abundance of the *Bacteroides* genus, indicating enhancement of the populations of SCFA-synthesizing bacteria, along with an enrichment of the anti-inflammatory bacterium *Duncaniella* (Yang et al. 2023). The LPS group exhibited a significant correlation with the levels of coenzyme Q2, which has been to promote the generation of mitochondrial ROS in the heart (Gharib et al. 2012). Furthermore, the metabolite cholic acid was differentially expressed between the LPS and Fisetin groups, with elevated levels in the former. This increase in cholic acid levels may have compromised the epithelial barrier, thereby promoting a shift toward pro-inflammatory gut microbiota (Chen et al. 2022). Collectively, the findings of this study suggest that FIS may mitigate mastitis by modulating gut barrier permeability, reducing inflammation, and increasing the levels of SCFAs, while simultaneously decreasing the concentrations of detrimental metabolites such as coenzyme Q2 and cholic acid.

However, there are still some limitations in this study, and we will gradually solve these problems in the subsequent experiments. First of all, although this study is based on animal and cell experiments, it may be different from the actual clinical situation. Subsequently, dairy cows will be used as experimental animals for further clinical trials. Secondly, this study will explore the therapeutic targets of fisetin on dairy cow mastitis in oxidative stress and ferroptosis signaling pathway through network pharmacology and protein interaction experiments, and further clarify its mechanism of action.

## 5. Conclusions

Our findings demonstrate that FIS significantly inhibits the production of ROS and malondialdehyde (MDA) and prevents the depletion of glutathione (GSH) in LPS-induced MAC-T cells, thereby restoring the total cellular antioxidant capacity. Additionally, it activates Nrf2 and xCT pathways to counteract LPS-induced ferroptosis. *In vivo*, LPS exposure induced ferroptosis in the mammary tissues of mice, as evidenced by increased total iron content and elevated inflammatory responses. This was accompanied by a reduction in SCFAs and anti-inflammatory bacterial species along with an increase in harmful metabolites. FIS treatment effectively reversed these changes, restoring the beneficial bacterial species ratio, increasing SCFAs levels, and reducing the levels of harmful metabolites. These findings suggest that FIS exerts protective effects against mastitis through redox modulation, ferroptosis inhibition, and microbiota regulation. Future research should focus on dose optimization, delivery methods, and validation in bovine models to explore its transformation potential in veterinary clinical settings.

## Supplementary Material

Graphical Abstract.tiff

## Data Availability

Sequence data that support the findings of this study have been deposited in the China National Center for Bioinformation, The GSA database is coded as CRA026762, and the OMIX database is coded as OMIX010591.

## References

[CIT0001] Ajose DJ et al. 2022. Combating bovine mastitis in the dairy sector in an era of antimicrobial resistance: ethno-veterinary medicinal option as a viable alternative approach. Front Vet Sci. 9:800322. 10.3389/fvets.2022.800322 35445101 PMC9014217

[CIT0002] Algammal AM, Enany ME, El-Tarabili RM, Ghobashy MOI, Helmy YA. 2020. Prevalence, antimicrobial resistance profiles, virulence and enterotoxins-determinant genes of MRSA isolated from subclinical bovine mastitis in Egypt, Pathog. Pathogens. 9(5):362. 10.3390/pathogens9050362 32397408 PMC7281566

[CIT0003] Angeli JPF et al. 2014. Inactivation of the ferroptosis regulator Gpx4 triggers acute renal failure in mice. Nat Cell Biol. 16:1180–1191. 10.1038/ncb3064 25402683 PMC4894846

[CIT0004] Chen L et al. 2022. Hepatic cytochrome P450 8B1 and cholic acid potentiate intestinal epithelial injury in colitis by suppressing intestinal stem cell renewal. Cell Stem Cell. 29(9):1366–1381.e9. 10.1016/j.stem.2022.08.008 36055192 PMC10673678

[CIT0005] Dinkova-Kostova AT, Talalay P. 2010. NAD(P)H:quinone acceptor oxidoreductase 1 (NQO1), a multifunctional antioxidant enzyme and exceptionally versatile cytoprotector. Arch Biochem Biophys. 501(1):116–123. 10.1016/j.abb.2010.03.019 20361926 PMC2930038

[CIT0006] Drummond GS, Baum J, Greenberg M, Lewis D, Abraham NG. 2019. HO-1 overexpression and underexpression: clinical implications. Arch Biochem Biophys. 673:108073. 10.1016/j.abb.2019.108073 31425676 PMC6748652

[CIT0007] Gerunova LK et al. 2024. Butyric acid and prospects for creation of new medicines based on its derivatives: a literature review. J Vet Sci. 25(2):e23. 10.4142/jvs.23230 38568825 PMC10990906

[CIT0008] Gharib A et al. 2012. Opposite and tissue-specific effects of coenzyme Q2 on mPTP opening and ROS production between heart and liver mitochondria: role of complex I. J Mol Cell Cardiol. 52(5):1091–1095. 10.1016/j.yjmcc.2012.02.005 22387164

[CIT0009] Harris IS, DeNicola GM. 2020. The complex interplay between antioxidants and ROS in cancer. Trend Cell Biol. 30(6):440–451. 10.1016/j.tcb.2020.03.002 32303435

[CIT0010] Heikkilä A-M, Nousiainen JI, Pyörälä S. 2012. Costs of clinical mastitis with special reference to premature culling. J Dairy Sci. 95(1):139–150. 10.3168/jds.2011-4321 22192193

[CIT0011] Hu X et al. 2020. The gut microbiota contributes to the development of staphylococcus aureus-induced mastitis in mice. Isme J. 14(7):1897–1910. 10.1038/s41396-020-0651-1 32341472 PMC7305118

[CIT0012] Hu X et al. 2022. The rumen microbiota contributes to the development of mastitis in dairy cows. Microbiol Spectr. 10(1):e02512-21. 10.1128/spectrum.02512-21 35196821 PMC8865570

[CIT0013] Jiang K, Yang J, Xue G, Dai A, Wu H. 2021. Fisetin ameliorates the inflammation and oxidative stress in lipopolysaccharide-induced endometritis. J Inflamm Res. 14:2963–2978. 10.2147/JIR.S314130 34262322 PMC8275103

[CIT0014] Kashyap D et al. 2019. Fisetin and quercetin: promising flavonoids with chemopreventive potential. Biomolecules. 9(5):174. 10.3390/biom9050174 31064104 PMC6572624

[CIT0015] Kløve DC, Jensen VF, Astrup LB. 2022. First finding of a methicillin-resistant staphylococcus aureus (MRSA) t304/ST6 from bovine clinical mastitis. Antibiotics (Basel, Switz.). 11(10):1393. 10.3390/antibiotics11101393 PMC9598757 36290051

[CIT0016] Kobayashi K, Oyama S, Numata A, Rahman M, Kumura H. 2013. Lipopolysaccharide disrupts the milk-blood barrier by modulating claudins in mammary alveolar tight junctions. PLoS One. 8(4):e62187. 10.1371/journal.pone.0062187 23626786 PMC3633878

[CIT0017] Koriem KMM, Farouk YKO. 2023. Fisetin treats kidney oxidative stress, inflammation, and apoptosis in rat diarrhea. Front Biosci (Sch. Ed.). 15:14. 10.31083/j.fbs1504014 38163954

[CIT0018] Kovačević Z et al. 2022. Pharmacoeconomic analysis of the different therapeutic approaches in control of bovine mastitis: phytotherapy and antimicrobial treatment. Antibiotics. 12(1):11. 10.3390/antibiotics12010011 36671213 PMC9854675

[CIT0019] Li D et al. 2021. Fisetin attenuates doxorubicin-induced cardiomyopathy In vivo and In vitro by inhibiting ferroptosis through SIRT1/Nrf2 signaling pathway activation. Front Pharmacol. 12:808480. 10.3389/fphar.2021.808480 35273493 PMC8902236

[CIT0020] Li R et al. 2021. Curcumin alleviates LPS-induced oxidative stress, inflammation and apoptosis in bovine mammary epithelial cells via the NFE2L2 signaling pathway. Toxins (Basel). 13(3):208. 10.3390/toxins13030208 33809242 PMC7999830

[CIT0021] Liu H, Lu Q. 2024. Fisetin alleviates inflammation and oxidative stress in deep vein thrombosis via MAPK and NRF2 signaling pathway. Int J Mol Sci. 25(7):3724. 10.3390/ijms25073724 38612535 PMC11011948

[CIT0022] Liu X et al. 2025. Alleviation of obesity cardiomyopathy by fisetin through the inhibition of NF-κB/MAPK signaling. Int Immunopharmacol. 151:114319. 10.1016/j.intimp.2025.114319 39983421

[CIT0023] Ma C et al. 2018. Cow-to-mouse fecal transplantations suggest intestinal microbiome as one cause of mastitis. Microbiome. 6(1):200. 10.1186/s40168-018-0578-1 30409169 PMC6225715

[CIT0024] Ma Z et al. 2021. Diverse β-lactam antibiotic-resistant bacteria and microbial community in milk from mastitic cows. Appl Microbiol Biotechnol. 105(5):2109–2121. 10.1007/s00253-021-11167-4 33587158

[CIT0025] Matić S, Stanić S, Mihailović M, Bogojević D. 2016. Cotinus coggygria scop.: an overview of its chemical constituents, pharmacological and toxicological potential. Saudi J Biol Sci. 23(4):452–461. 10.1016/j.sjbs.2015.05.012 27298577 PMC4890191

[CIT0026] Maurya BK, Trigun SK. 2016. Fisetin modulates antioxidant enzymes and inflammatory factors to inhibit aflatoxin-B1 induced hepatocellular carcinoma in rats. Oxid Med Cell Longev. 2016(1):1972793. () 10.1155/2016/1972793 26682000 PMC4670673

[CIT0027] Meng M et al. 2022. Lentinan inhibits oxidative stress and alleviates LPS-induced inflammation and apoptosis of BMECs by activating the Nrf2 signaling pathway. Int J Biol Macromol. 222(Pt B):2375–2391. 10.1016/j.ijbiomac.2022.10.024 36243161

[CIT0028] Mushtaq S et al. 2018. Bovine mastitis: an appraisal of its alternative herbal cure. Microb Pathog. 114:357–361. 10.1016/j.micpath.2017.12.024 29233776

[CIT0029] Paramasivam R et al. 2023. Is AMR in dairy products a threat to human health? An updated review on the origin, prevention, treatment, and economic impacts of subclinical mastitis. Infect Drug Resist. 16:155–178. 10.2147/IDR.S384776 36636377 PMC9831082

[CIT0030] Park C et al. 2023. Fisetin protects C2C12 mouse myoblasts from oxidative stress-induced cytotoxicity through regulation of the Nrf2/HO-1 signaling. J Microbiol Biotechnol. 33(5):591–599. 10.4014/jmb.2212.12042 36859395 PMC10236176

[CIT0031] Qadri H et al. 2022. Natural products and their semi-synthetic derivatives against antimicrobial-resistant human pathogenic bacteria and fungi. Saudi J Biol Sci. 29(9):103376. 10.1016/j.sjbs.2022.103376 35874656 PMC9290337

[CIT0032] Qian X et al. 2023. Fisetin ameliorates diabetic nephropathy-induced podocyte injury by modulating Nrf2/HO-1/GPX4 signaling pathway, Evid.-Based Complement. Altern. Med Ecam. 9331546. 10.1155/2023/9331546 PMC11401708 39281805

[CIT0033] Romero-Durán MA, Silva-García O, Perez-Aguilar JM, Baizabal-Aguirre VM. 2024. Mechanisms of Keap1/Nrf2 modulation in bacterial infections: implications in persistence and clearance. Front Immunol. 15:1508787. 10.3389/fimmu.2024.1508787 39763664 PMC11700987

[CIT0034] Sadat A et al. 2023. Immunological and oxidative biomarkers in bovine serum from healthy, clinical, and sub-clinical mastitis caused by escherichia coli and staphylococcus aureus infection. Anim Open Access J MDPI. 13(5):892. 10.3390/ani13050892 PMC10000043 36899749

[CIT0035] Sindhu S et al. 2024. Beyond conventional antibiotics approaches: global perspectives on alternative therapeutics including herbal prevention, and proactive management strategies in bovine mastitis. Microb Pathog. 196:106989. 10.1016/j.micpath.2024.106989 39357684

[CIT0036] Suganya T et al. 2022. Tackling multiple-drug-resistant bacteria with conventional and complex phytochemicals. Front Cell Infect Microbiol. 12:883839. 10.3389/fcimb.2022.883839 35846771 PMC9280687

[CIT0037] Sukhikh S et al. 2021. Study of the biologically active properties of medicinal plant Cotinus coggygria. Plants (Basel). 10(6):1224. 10.3390/plants10061224 34208532 PMC8235186

[CIT0038] Sun M et al. 2025. Stigmasterol from prunella vulgaris L. Alleviates LPS-induced mammary gland injury by inhibiting inflammation and ferroptosis. Phytomedicine. 137:156362. 10.1016/j.phymed.2025.156362 39809030

[CIT0039] Tonelli C, Chio IIC, Tuveson DA. 2018. Transcriptional regulation by Nrf2. Antioxid Redox Signal. 29(17):1727–1745. 10.1089/ars.2017.7342 28899199 PMC6208165

[CIT0040] Tong X et al. 2025. Virulence of bacteria causing mastitis in dairy cows: a literature review. Microorganisms. 13(1):167. 10.3390/microorganisms13010167 39858935 PMC11767654

[CIT0041] Wang B et al. 2024. Fisetin ameliorates fibrotic kidney disease in mice via inhibiting ACSL4-mediated tubular ferroptosis. Acta Pharmacol Sin. 45(1):150–165. 10.1038/s41401-023-01156-w 37696989 PMC10770410

[CIT0042] Wang X et al. 2023. Resveratrol reduces ROS-induced ferroptosis by activating SIRT3 and compensating the GSH/GPX4 pathway. Mol Med. 29(1):137. 10.1186/s10020-023-00730-6 37858064 PMC10588250

[CIT0043] Wang Y et al. 2024. Nutrition, gastrointestinal microorganisms and metabolites in mastitis occurrence and control. Anim Nutr. 17:220–231. 10.1016/j.aninu.2024.01.010 38800734 PMC11126769

[CIT0044] Yang H et al. 2024. Fisetin exerts neuroprotective effects in vivo and in vitro by inhibiting ferroptosis and oxidative stress after traumatic brain injury. Front. Pharmacol. 15:1480345. 10.3389/fphar.2024.1480345 39635435 PMC11615404

[CIT0045] Yang M, Han J, Yan Z, Li K. 2025. Paeonol attenuates LPS-induced inflammatory injury in mastitis by regulating the MAPK and Nrf2 signaling pathways. Res Vet Sci. 182:105481. 10.1016/j.rvsc.2024.105481 39608062

[CIT0046] Yang S, Lian G. 2020. ROS and diseases: role in metabolism and energy supply. Mol Cell Biochem. 467(1-2):1–12. 10.1007/s11010-019-03667-9 31813106 PMC7089381

[CIT0047] Yang W et al. 2022. Maresin1 protect against ferroptosis-induced liver injury through ROS inhibition and Nrf2/HO-1/GPX4 activation. Front Pharmacol. 13:865689. 10.3389/fphar.2022.865689 35444546 PMC9013935

[CIT0048] Yang Y, Zhao X, Xie Y, Wu C. 2023. Modulative effect of physalis alkekengi on both gut bacterial and fungal micro-ecosystem. Chin Herb Med. 15(4):564–573. 10.1016/j.chmed.2023.02.003 38094014 PMC10715876

[CIT0049] Zaatout N. 2022. An overview on mastitis-associated Escherichia coli: pathogenicity, host immunity and the use of alternative therapies. Microbiol Res. 256:126960. 10.1016/j.micres.2021.126960 35021119

[CIT0050] Zhu J et al. 2023. The deubiquitinase USP11 ameliorates intervertebral disc degeneration by regulating oxidative stress-induced ferroptosis via deubiquitinating and stabilizing Sirt3. Redox Biol. 62:102707. 10.1016/j.redox.2023.102707 37099926 PMC10149406

[CIT0051] Zhuang C et al. 2024. Escherichia coli infection induces ferroptosis in bovine mammary epithelial cells by activating the wnt/β-catenin pathway-mediated mitophagy. Mitochondrion. 78:101921. 10.1016/j.mito.2024.101921 38885732

