# Supplementary figures and images for: Fisetin alleviates lipopolysaccharide-induced mastitis by inhibiting ferroptosis and modulating the gut microbiota
Source: Vet Q. 2026 Mar 17;46(1):2642789. doi: 10.1080/01652176.2026.2642789 (PMC13003875; doi:10.1080/01652176.2026.2642789)

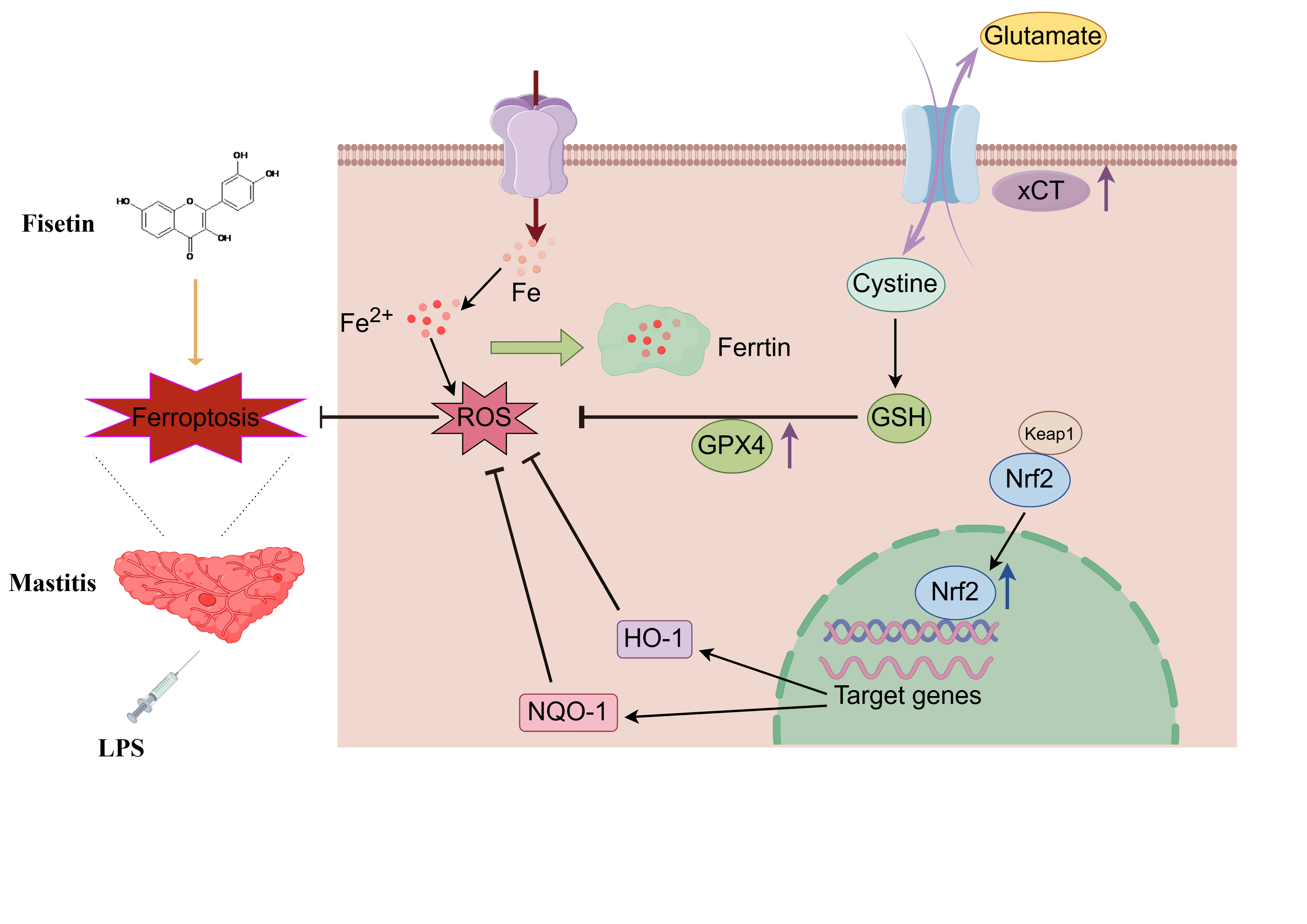

Supplement: Graphical Abstract.tiff [file TVEQ_A_2642789_SM7801.tiff]
